# Association of Metabolic Syndrome and Stroke in Adults: A Case-Control Study

**DOI:** 10.7759/cureus.91615

**Published:** 2025-09-04

**Authors:** Satya S Tripathy, Girija Shankar P Patro, Subrat K Pradhan, Sanjeeb K Mishra

**Affiliations:** 1 Community Medicine, Veer Surendra Sai Institute of Medical Sciences and Research, Sambalpur, IND; 2 Anaesthesiology, Bhima Bhoi Medical College and Hospital, Bolangir, IND; 3 Field Epidemiology Training Program, Indian Council of Medical Research, Chennai, IND; 4 Community Medicine, Veer Surendra Sai Institute of Medical Sciences And Research, Sambalpur, IND

**Keywords:** cardiometabolic risk factors, case-control studies, logistic models, metabolic syndrome, odds ratio, stroke

## Abstract

Background

Stroke is a global health issue and a major contributor to morbidity and mortality. In India, the incidence rate of stroke is on a rising trend. Metabolic syndrome has close associations with the pathogenesis of stroke. Evidence of the association between various other risk factors and stroke is scarce in developing countries like India. This study aims to determine the association between metabolic syndrome and stroke.

Methodology

An unmatched case-control study was conducted at Veer Surendra Sai Institute of Medical Sciences And Research (VIMSAR), Burla, Odisha, India, taking 56 cases of stroke and 56 controls without stroke from September 2022 to August 2024. The study population included patients admitted to the Department of Internal Medicine. Study participants were selected randomly from the In-Patient Department thrice weekly. Data were collected using a predesigned, pre-tested questionnaire. Data were analysed using SPSS v. 21 (IBM Corp., Armonk, NY, US). Proportions, the chi-square test, and binary logistic regression were applied to arrive at the final interpretation.

Results

Of 112 study participants, 71 (63.4%) were males and the rest were females. The median age in cases was 62.5 (70, 56) and 50 (60, 38.5) in controls. Metabolic syndrome (adjusted odds ratio (AOR) = 2.8), salt intake >1 teaspoon per day (AOR = 10.0), age > 60 years (AOR = 5.3), BMI ≥ 25 kg/m^2^ (AOR = 4.6), and smoking/chewing of tobacco (AOR = 3.0) were found to be significant risk factors of stroke. No significant association was found between stroke and factors such as gender, area of residence, caste, socioeconomic status, consumption of alcohol, consumption of fruits and vegetables, and exercise.

Conclusion

Our study has once again ascertained metabolic syndrome as a significant risk factor for stroke. We also found modifiable risk factors like high salt intake, BMI, and consumption of tobacco to be significant predictors of stroke. These risk factors can be addressed during non-communicable disease (NCD) screening so that people at risk of stroke can be identified at an early stage, which, in turn, can minimize the risk and complications due to stroke later in life.

## Introduction

The World Health Organization defines stroke as “rapidly developing clinical signs of focal or global disturbance of cerebral function, lasting more than 24 hours or until death, with no apparent non-vascular cause” [1]. Stroke is a major public health concern, contributing significantly to both morbidity and mortality. According to the Global Burden of Disease (GBD) 2019 estimates, stroke was the second leading cause of death globally (6.55 million deaths) and the third leading cause of death and disability combined, with 12.2 million incident cases reported in 2019 [2].

In India, there has been an increasing incidence of stroke from 1990 to 2019. In 2019 alone, the estimated number of incident stroke cases in India was 1.29 million (95% uncertainty interval (UI): 1.15-1.45 million), with 699,000 deaths attributed to stroke (95% UI: 594,000-807,000) [3]. Nearly 90% of the global stroke burden occurs in low- and middle-income countries (LMICs) and among younger populations [4].

Metabolic syndrome (MetS) is closely linked to the pathogenesis of stroke. This syndrome comprises a cluster of metabolic abnormalities--atherogenic dyslipidaemia, hypertension, insulin resistance, and obesity--that are common risk factors for both stroke and MetS. A diagnosis of metabolic syndrome is made when an individual presents with three or more of the following conditions: high blood glucose, low levels of high-density lipoproteins (HDL), elevated total cholesterol, high triglyceride levels, elevated body mass index (BMI), and high blood pressure [5]. In India, the prevalence of metabolic syndrome among adults is approximately 30%. Notably, the prevalence increases steadily with age, from 13% among those aged 18-29 years to 50% in the 50-59-year age group [6]. According to GBD estimates, metabolic risk factors account for 71.0% of the global stroke burden. These risk factors include elevated blood pressure, high BMI, hyperglycaemia, and hyperlipidaemia [4]. Several studies have reported a higher prevalence of MetS among stroke patients compared to control groups [7,8]. Both stroke and MetS show an increasing trend and share similar underlying metabolic risk factors.

Metabolic syndrome has significant implications for individual health outcomes and healthcare costs. Therefore, research examining the relationship between stroke and MetS, particularly in this region of India, is of significant public health importance. It will help researchers, epidemiologists, and policymakers develop more effective strategies for the prevention and control of these major non-communicable diseases (NCDs). In this context, the primary objective of the study was to determine whether metabolic syndrome is associated with ischemic stroke among adults admitted to Veer Surendra Sai Institute of Medical Sciences and Research (VIMSAR), Burla, India.

## Materials and methods

Study design

This unmatched case-control study was conducted among 56 cases and 56 controls that fulfilled the inclusion and exclusion criteria and were admitted to the In-Patients Department (IPD) of the Department of Internal Medicine of Veer Surendra Sai Institute of Medical Sciences and Research (VIMSAR), Burla, Sambalpur, Odisha, India, from September 2022 to August 2024.

Selection criteria

Patients aged more than 30 years of age, hospitalized and diagnosed with ischemic stroke based on clinical findings and confirmed by a CT scan brain, willing to participate in the study, and present at the time of data collection were included as cases in the study. Patients who were suffering from stroke due to trauma or embolic stroke were excluded. Patients aged more than 30 years and not suffering from stroke or without a prior history of stroke were included as controls. Critically ill patients were excluded as controls from the study.

Sampling

Cases for data collection were sourced on a thrice-weekly basis. On each day of data collection, a maximum of four stroke patients satisfying the inclusion criteria were randomly selected from the IPD register and enrolled as study subjects. Controls were selected from the IPD register on a thrice-weekly basis. An equal number of controls were enrolled on each day of data collection. Controls were selected randomly from the register in the IPD.

The interview questionnaire included socio-demographic factors, lifestyle profile, physical profile, clinical profile, and biochemical profile (Appendices). A stroke is defined as an acute onset of focal neurological deficit that is attributed to a vascular cause that lasts for more than 24 hours. The American Heart Association (AHA)/National Heart, Lung, and Blood Institute (NHLBI) proposed a set of criteria for the diagnosis of metabolic syndrome in 2005. This required fulfilment of any 3 of the following 5 elements: increased waist circumference (≥ 40 inches in males and ≥ 35 inches in females), fasting triglyceride level ≥ 150 mg/dl, HDL-C <40 mg/dl in males and < 50 mg/dl in females, elevated blood pressure ≥130/85 mm Hg and elevated fasting glucose >100 mg/dl [9].

To reduce recall bias, it was ensured that participants were unaware of the hypothesis being tested, and a proper interview schedule was used. Only Medicine IPD-registered cases were interviewed during the data collection period to minimize selection bias, and unavailable patients and those who left against medical advice were excluded from the study. Logistic regression was used to control multiple confounders. The odds ratio thus obtained (adjusted odds ratio (AOR)) was adjusted for other covariates, including confounders.

Sample size

The sample size for this study was calculated using OpenEpi, a free and open-source statistical software program designed for public health professionals by the Centers for Disease Control and Prevention (CDC) (www.OpenEpi.com). Assuming metabolic syndrome as a risk factor with the lowest odds ratio of 3.17 and 34% among controls and 62% among cases from the literature reviewed [7], with 95% confidence interval assumptions and 5% marginal error with 80% power. The ratio for cases to controls was assumed to be 1. The calculated sample size was 112.

Data collection

A pre-tested structured questionnaire was used to collect data from each study participant (Appendices). This questionnaire was also used to collect data from patient bedhead tickets: interviews collected socio-demographic, lifestyle, and a few clinical characteristics information. Anthropometric data, including height, waist circumference, and weight, were measured using a standardized non-stretchable tape and a weighing scale. Those patients who could stand were made to stand against the wall, and a ruler was placed on top of their head parallel to the ground, and the height was marked on the wall using a marker. The height of the patients who could not stand was recorded by measuring the arm span. Blood pressure was measured using a sphygmomanometer. The sphygmomanometer was placed on a horizontal surface at the person’s heart level, and the mercury column was at the observer’s eye level. BP was measured first by the palpatory method and then by the auscultatory method. Biochemical profiling was done on the hospital premises in the Regional Diagnostic Centre (RDC).

Data entry and analysis

At the end of the interview, all the data collected were crosschecked to ensure the completeness and accuracy of the data and that there was no missing data. Statistical Package for the Social Sciences (SPSS) v. 21 (IBM Corp., Armonk, NY, US) was used for data analysis. Data entry was done after data collection from cases and controls, after coding the different variables into the relevant categories.

Descriptive analysis was done based on the frequency distribution of selected socio-demographic characteristics. The median and IQR of selected study variables were obtained. Dummy tables were used to guide the analysis. The respondents were categorized into those with ischemic stroke (cases) and those without ischemic stroke (controls). The crude odds ratio (COR), the chi-square test for categorical variables at a 95% confidence interval (CI), and an alpha level of significance set at 0.05 were used as measures of association in analyzing factors associated with stroke. An odds ratio of > 1 was considered a risk factor. A confidence interval was used to assess the variability of the odds ratio. The final analysis was done using binary logistic regression for those factors with a p-value of < 0.05, and the backward elimination method was used to determine the factors that were independent predictors of stroke. The final model contained only statistically significant factors after adjusting for confounding.

## Results

Among the cases, 37 (66%) were males, and 34 (60.7%) were males in controls. The median age of participants in cases was 62.5 years with an inter-quartile range (IQR) of 14 (70, 56) years, which was higher than that of the control group with a median age of 50 years and an IQR of 21.5 (60, 38.5) years.

Table 1 shows the association between various factors and stroke. Among cases, the elderly (>60 years) age group was the majority, whereas the 30-60 age group predominated among controls. Rural inhabitation was higher among cases as compared to controls, where the majority resided in urban areas. Among cases, the scheduled caste category was the majority. The general category was the majority among controls, followed by the scheduled caste, scheduled tribe, and other backward classes. Most study participants belonged to the lower middle socio-economic category, i.e., 37 (66%) and 32 (57.1%) in cases and controls, respectively. The lowest number was from the upper and lower socio-economic status. Most of the stroke patients were tobacco users (smoking or chewing). The number of tobacco users was 21 (37.5%) among controls. About 14 (25%) cases and 15 (26.8%) controls consumed alcohol. About 42 (75%) stroke patients and 28 (50%) controls didn’t exercise. Nearly 16 (28.6%) stroke patients and 19 (33.9%) controls consumed the recommended quantity of fruits and vegetables. About 48 (85.7%) stroke cases consumed > 1 teaspoon (5-6 grams) salt per day, whereas in controls, most consumed within the limits.

**Table 1:** Association of various factors with stroke *-Represents a significant p-value, as it is <0.05 SC: scheduled caste; ST; scheduled tribe; OBC: other backward tribe

Sl. No.	Characteristic Feature		Cases (%), n=56	Control (%), n=56	Chi-square value	P value
1	Gender	Male	37 (66)	34 (60.7)	0.346	0.556
		Female	19 (34)	22 (39.3)		
2	Age group	30-60	24 (42.9)	46 (82.1)	18.438	<0.0001*
		>60	32 (57.1)	10 (17.9)		
3	Residence	Rural	33 (58.9)	25 (44.6)	2.289	0.13
		Urban	23 (41.1)	31 (55.4)		
5	Caste	SC	24 (42.8)	13 (23.2)	5.662	0.129
		ST	10 (17.9)	13 (23.2)		
		OBC	10 (17.9)	10 (17.9)		
		General	12 (21.4)	20 (35.7)		
7	Socio-economic status (Modified Kuppuswamy Scale)	Upper middle	14 (25)	22 (39.3)	3.426	0.18
		Lower middle	37 (66)	32 (57.1)		
		Upper lower	5 (9)	2 (3.6)		
8	Smoke/Chew tobacco	Yes	36 (64.3)	21 (37.5)	8.038	0.005*
		No	20 (35.7)	35 (62.5)		
9	Consume alcohol	Yes	14 (25)	15 (26.8)	0.047	0.829
		No	42 (75)	41 (73.2)		
10	Exercise	Yes	14 (25)	28 (50)	7.467	0.006*
		No	42 (75)	28 (50)		
11	Consumption of fruits and vegetables	One bowl (100 gms)	10 (17.8)	2 (3.6)	5.975	0.05
		Two bowls (200 gms)	30 (53.6)	35 (62.5)		
		Three bowls (300 gms)	16 (28.6)	19 (33.9)		
12	Salt intake	≤1 teaspoon/day	8 (14.3)	32 (57.1)	22.400	<0.0001*
		>1 teaspoon/day	48 (85.7)	24 (42.9)		
13	BMI	≥25	39 (69.6)	19 (34)	14.304	<0.0001*
		<25	17 (30.4)	37 (66)		
14	Metabolic syndrome	Yes	38 (67.9)	21 (37.5)	10.351	0.001*
		No	18 (32.1)	35 (62.5)		

Nearly 39 (69.6%) stroke patients and 19 (34%) controls had a BMI of ≥ 25 kg/m^2^. The majority of the cases were suffering from metabolic syndrome, as opposed to just 21 (37.5%) among controls. On statistical analysis, age category, i.e., 30-60 and > 60 years, was found to have a significant association with the occurrence of stroke, with a value less than 0.0001 and a chi-square value of 18.438. Use of tobacco, dedicated hours of exercise (other than routine activity), and salt intake were found to be significantly associated (p< 0.05) with stroke (chi-square value of 8.038, 7.467, and 22.4, respectively, and degree of freedom 1). BMI and metabolic syndrome had a significant association with stroke, with chi-square values of 14.403 and 10.351 and p-values of < 0.0001 and 0.001.

Factors with a p-value of < 0.05 during the chi-square test were entered into a binary logistic regression model simultaneously (backward conditional logistic regression), except for the components of metabolic syndrome, and the risk factors were analyzed.

Table 2 shows a binary logistic regression analysis of study participants. Participants with metabolic syndrome had a 2.8 times higher risk of suffering from ischemic stroke. Salt intake of more than 1 teaspoon per day increases the odds of causing stroke 10.0 times more than those who consume ≤ 1 teaspoon per day. BMI ≥ 25 kg/m^2^ (AOR = 4.6) was found to be significantly associated with stroke. A group of participants belonging to the age group of > 60 years of age was found to be at a 5.3 times higher risk than those between the ages of 30 and 60 years of getting a stroke. Study participants who consumed tobacco in either the form of smoking or chewing were at a 3.0 times higher risk of suffering from stroke than those who didn’t consume any. Exercise other than routine activity was not significantly associated with stroke after binary logistic regression. The binary logistic regression model was significant with chi-square 59.162, degree of freedom 5, and p-value < 0.0001. The model explained 54.7% Nagelkerke R^2^ of the variance in stroke and correctly classified 80.4% of the cases.

**Table 2:** Binary logistic regression analysis *- Represents a significant p-value, as it is <0.05 COR: crude odds ratio; CI: confidence interval; AOR: adjusted odds ratio

Sl no.	Factor		Case (%), n=56	Control (%), n=56	COR (95% C.I.)	AOR (95% C.I.)	P value
1	Metabolic syndrome	Yes	38 (67.9)	21 (37.5)	3.5 (1.6- 7.6)	2.8 (1.0- 8.0)	0.046*
		No	18 (32.1)	35 (62.5)			
2	Salt intake	>1 teaspoon/ day	48 (85.7)	24 (42.9)	8.0 (3.1- 20.0)	10.0 (2.9- 33.7)	<0.0001*
		≤1 teaspoon/ day	8 (14.3)	32 (57.1)			
3	Age group	>60	32 (57.1)	10 (17.9)	6.1 (2.5- 14.5)	5.3 (1.8- 15.2)	0.002*
		30-60	24 (42.9)	46 (82.1)			
4	BMI	≥25	39 (69.6)	19 (34)	4.4 (2.0- 9.8)	4.6 (1.6- 13.3)	0.004*
		<25	17 (30.4)	37 (66)			
5	Smoke/Chew tobacco	Yes	36 (64.3)	21 (37.5)	3.0 (1.3- 6.4)	3.0 (1.0- 8.2)	0.033*
		No	20 (35.7)	35 (62.5)			
6	Exercise	No	42 (75)	28 (50)	3.0 (1.3- 6.6)	1.5 (0.4- 4.6)	0.487
		Yes	14 (25)	28 (50)			

## Discussion

Our study was an unmatched case-control study conducted among 112 participants to examine the association between MetS and stroke in adults. The presence of MetS (AOR: 2.8), salt intake of more than 1 teaspoon per day (AOR: 10.0), age above 60 years (AOR: 5.3), BMI ≥ 25 kg/m^2^ (AOR: 4.7), and tobacco use (smoking or chewing; AOR: 3.0) were identified as significant risk factors for stroke. Among the cases, 37 (66%) were male, as compared to 34 (60.7%) in the control group. No significant association was found between gender and stroke. However, studies by Akyea RK et al. and Bai X et al. reported a higher incidence of stroke in women as compared to men [10,11].

In our study, 32 (57.2%) cases and 10 (17.9%) controls were aged over 60 years, with the remainder falling in the 30-60-year age group. Participants aged above 60 years showed a significant association with stroke (AOR: 5.3, 95% CI: 1.845-15.277). Akyea RK et al. reported similar findings, with a mean age of stroke onset being 74.4 years [10]. The area of residence--rural or urban--showed no significant association with stroke in our study.

Tobacco use (smoking or chewing) was significantly associated with stroke (AOR: 3.0, 95% CI: 1.092-8.276). Tobacco users had a threefold increased risk of stroke compared to non-users. Similar findings were reported by Rohr J et al., with odds ratios of 2.0 and 3.3 for white and black men, respectively, and 2.1 and 2.2 for white and black women, respectively [12]. A study by You RX et al. also found current smoking to be a significant risk factor for stroke among adults aged 15-55 years (OR: 2.5) [13].

Alcohol consumption did not show a significant association with ischemic stroke in our study. In contrast, You RX et al. reported that long-term heavy alcohol consumption (>60 g/day) was associated with a 15.3-fold increased risk of stroke, whereas recent heavy drinking within the preceding 24 hours was not a significant risk factor [13]. Smyth A et al. found that high alcohol intake significantly increased the risk of ischemic stroke (OR: 1.55), while low intake (OR: 0.98) and current drinking (OR: 1.06) were not significantly associated [14]. Djoussé L et al. reported that former drinkers consuming ≥ 12 g/day had a 2.4-fold increased risk of ischemic stroke in men, but not in women [15].

Physical inactivity was significantly associated with stroke in unadjusted analysis (COR: 3.0, 95% CI: 1.3-6.6), but not in the adjusted model (AOR: 1.5, 95% CI: 0.4-4.6). Guo J et al. found that physical inactivity significantly increased ischemic stroke risk in women with diabetes (OR: 1.57), but not in men [16]. Differences in sample size and study populations may account for the discrepancies in findings. Fruit and vegetable consumption did not show a significant association with stroke in our study. In contrast, He FJ et al. found a reduced risk of stroke with 3-5 servings (RR: 0.89) and >5 servings (RR: 0.74) per day as compared to <3 servings/day [17]. Hu D et al. reported a multivariable RR of 0.79 for the highest vs. the lowest fruit and vegetable intake categories [18]. The differences may stem from the potential recall bias in our study, as most participants reported consuming around two bowls (200 g) of fruits and vegetables combined daily, and many did not consume fruits regularly.

Participants consuming more than 1 teaspoon (5-6 grams) of salt per day had a 10-fold higher risk of stroke than those consuming 1 teaspoon or less. This aligns with findings from Strazzullo P et al., who reported a pooled RR of 1.23 for salt intake > 6 g/day [19], and Li XY et al., who found that high salt intake was significantly associated with ischemic stroke mortality (OR: 2.15) but not stroke onset (OR: 1.07) [20].

A BMI ≥25 kg/m² was significantly associated with ischemic stroke in our study (AOR: 4.6, 95% CI: 1.6-13.3). Kroll ME et al. reported a relative risk of 1.21 per 5 kg/m² increase in BMI for ischemic stroke [21]. Bazzano LA et al. observed that among Chinese individuals, stroke risk increased with BMI, with relative risks of 1.43 for BMI 25-29.9 and 1.72 for BMI ≥30 [22]. Liu X et al. found increased stroke risk only at BMI >25 kg/m² [23]. Lu Y et al. also reported a significant hazard ratio of 1.18 per 5 kg/m² increase in BMI, with both overweight and obesity associated with higher risks of coronary heart disease and stroke [24].

Our study found metabolic syndrome to be significantly associated with stroke, with affected participants facing a 2.8-fold increased risk. This finding aligns with Ahmadi AM et al., who reported an OR of 2.9 [25], and Ashtari F et al., who reported an OR of 4.3 and found higher MetS prevalence in women [7]. Zhang F et al. found that low HDL levels (RR: 1.32), ≥ 2 MetS components (RR: 1.68), and MetS itself (RR: 1.46) significantly predicted stroke recurrence and mortality (RR: 1.27) [26]. A meta-analysis by Li X et al. also reported a pooled RR of 1.7 for incident stroke in participants with MetS [27]. Ninomiya JK et al. (OR: 2.16), Kurl S et al. (OR: 2.41), and Mottillo S et al. (RR: 2.27) all reported similar associations [28-30].

Limitations

Being an unmatched case-control design, this study could not establish temporality. Additionally, matching cases and controls could have helped control for known confounders. A larger sample size may have enabled the detection of more associations. Furthermore, dietary data were obtained through recall, which is susceptible to reporting bias and may have led to under- or overestimation of certain risk factors.

## Conclusions

This study reaffirmed that patients with metabolic syndrome (OR=3.5) had significantly higher odds of developing stroke than others. We also conclude that age > 60 years, salt intake > 1 teaspoon/day, BMI ≥ 25kg/m^2^, smoking/chewing tobacco, and no exercise except routine activity also significantly affected the risk of stroke. On binary logistic regression, the associations were confirmed, except for lack of exercise. The above risk factors may be used to screen the population at risk during routine checkups in sub-centers and primary and secondary health centers to identify people at risk of stroke early. As these are all modifiable with proper health education and lifestyle changes, interventions aimed at these can significantly reduce the morbidity and mortality due to stroke.

## Appendices

**Table 3:** Questionnaire

Serial number	Question	Response
1	Suffering from stroke?	Yes
		No
2	Name	
3	Age (completed years)	
4	Residence	Rural
		Urban
5	Gender	Male
		Female
6	Religion	Hindu
		Muslim
		Christian
		Others
7	Caste	SC
		ST
		OBC
		General
8	Occupation	Legislators, Senior Officials & Managers
		Professionals
		Technicians and Associate Professionals
		Clerks
		Skilled Workers and Shop & Market Sales Workers
		Skilled Agricultural & Fishery Workers
		Craft & Related Trade Workers
		Plant & Machine Operators and Assemblers
		Elementary Occupation
		Unemployed/ Homemaker
9	Educational status	Graduate
		Intermediate or diploma
		High school certificate
		Middle school certificate
		Primary school certificate
		Illiterate
10	Monthly Income	≥184,376
		92191- 184,370
		68967- 92185
		46095- 68961
		27654- 46089
		9232- 27648
		≤ 9226
11	Socio-economic status	Upper
		Upper Middle
		Lower Middle
		Upper Lower
		Lower
12	Do you smoke or chew tobacco?	Yes
		No
13	Do you consume alcohol?	Yes
		No
14	Do you exercise other than routine activity?	Yes
		No
15	On an average, how many servings of fruits and vegetables do you consume daily?	One bowl (100gms)
		Two bowls (200gms)
		Three bowls (300gms)
		More than three bowls (> 300gms)
16	Salt intake	≤1 Teaspoon/day
		>1 Teaspoon/day
17	Height (in m)	
18	Weight (in kg)	
19	BMI (kg/m^2^)	
20	Waist circumference (in inches)	
21	Pulse rate	
22	Blood Pressure	
23	Diabetes Mellitus	Present
		Absent
24	Hypertension	Present
		Absent
25	Dyslipidaemia	Present
		Absent
26	Fasting Triglyceride level	
27	HDL level	
28	FBS level	
29	Waist circumference (>40-inch Male, >35-inch Female)	Yes
		No
30	Blood pressure (>130/85 mm of Hg)	Yes
		No
31	Fasting Triglyceride level (>150 mg/dl)	Yes
		No
32	HDL level (<40 mg/dl male, <50 mg/dl female)	Yes
		No
33	FBS level (>100 mg/dl)	Yes
		No
34	Criteria for Metabolic Syndrome fulfilled?	Yes
		No
